# Chemical Peel Treats Nodular Lesions Following Mesotherapy: A Report of Three Cases

**DOI:** 10.1111/jocd.16785

**Published:** 2025-02-07

**Authors:** Siru Wu, Yanli Tian

**Affiliations:** ^1^ ADD^+^ Medical Esthetic Clinic Beijing China

To the editor,

As mesotherapy becomes increasingly popular, there is growing attention to nodular skin lesions after treatment. The persistent occurrence of these lesions may lead to the formation of foreign body granulomas. Here, we report three cases where chemical peel effectively relieved persistent nodular lesions following mesotherapy.

## 1 Case 1

**FIGURE 1:**
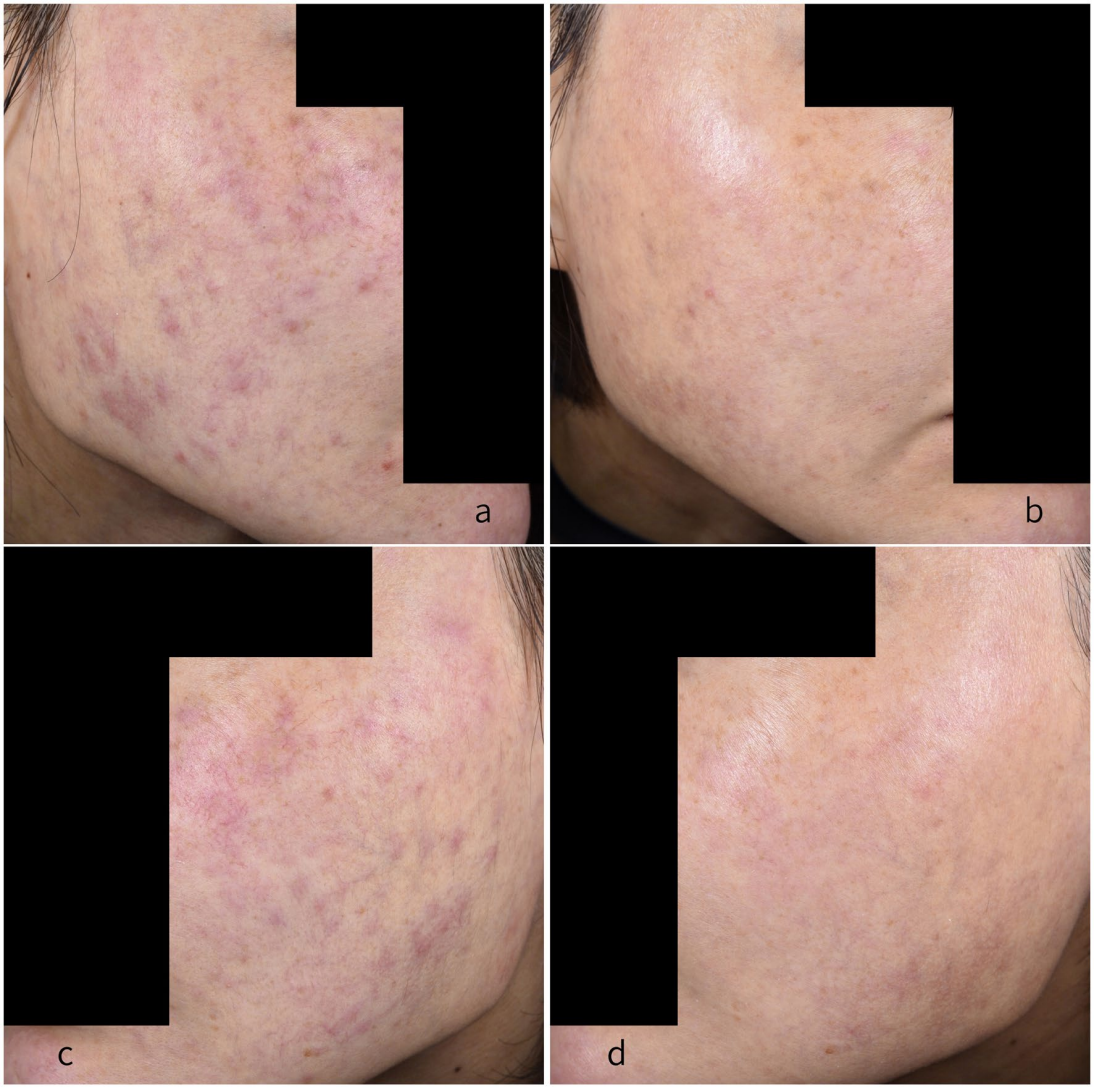
(a, c) Before treatment and (b, d) after treatment.

A 41‐year‐old female received intradermal injections of “collagen product” at a clinic for face rejuvenation. Within 1 week, erythema, edema, itching, and a burning sensation appeared on her face, followed by well‐defined and evenly distributed red nodules at the injection sites. Oral antibiotics and methylprednisolone (20 mg) per day were given at another clinic. The lesions subsided gradually but recurred after the discontinuation of the medication. After 15 days of injection, microneedling combined with 30% supramolecular salicylic acid (SSA)chemical peel (Broda, Shanghai Rui Zhi Medicine Technology) was performed at our clinic. The lesions gradually subsided and then resolved completely at the 1‐month follow‐up after three treatments (Figure 1).

## 2 Case 2

A 38‐year‐old female received intradermal injections of “collagen products” at a clinic for face rejuvenation. Shortly after the treatment, erythema, edema, and a burning sensation developed at the injection sites, followed by well‐defined red nodules. She was given oral antihistamine and methylprednisolone (24 mg) per day at another clinic. The edema subsided, but nodules remained prominent. One month after the injections, she received microneedling combined with 30% SSA therapy at our clinic. The lesions completely resolved at the 3‐month follow‐up after three sessions of combined treatments.

## 3 Case 3

A 28‐year‐old female received intradermal injections of “collagen products” at a clinic for neck rejuvenation. Three days after the treatment, well‐defined red nodules accompanied by itching developed at some injection sites. She was given oral antihistamines, prednisone (10 mg), and topical hydrocortisone butyrate cream per day at another clinic. Some of the lesions subsided gradually but recurred after discontinuing the medication. Thirty‐five days after the mesotherapy, the aforementioned treatment was performed at our clinic. The lesions subsided significantly at the 1‐month follow‐up after three treatments. She was unable to continue the treatment due to traveling abroad.

It is widely known that the adverse reaction of nodular lesions can be treated with systemic or intralesional corticosteroids or surgical excision [1, 2]. However, the use of these methods is often limited, as patients may refuse long‐term medication use due to concerns about its adverse effects or they may refuse intralesional therapy due to fears of local atrophy. Additionally, surgical excision may not be suitable for cases with numerous or widely distributed lesions. As a result, attention has gradually shifted to physical therapies, such as radiofrequency, ultrasound, and hyperbaric oxygen therapy [3, 4, 5]. There are only a few cases reported potentially because the above‐mentioned devices are hard to access.

The main aims of such treatments are anti‐inflammatory and anti‐hyperplasia [6]. NF‐κB is crucial to the mediation of innate immunity, adaptive immunity, and pro‐inflammatory responses. Functioning as a mediator in pro‐inflammatory responses, NF‐κB enhances macrophage activation and increases the release of IL‐1, IL‐6, IL‐12, TNF‐α, and other cytokines during chronic inflammation [7]. Liu et al. [8] suggest that their treatment may attenuate *Schistosoma. japonicum* egg‐induced hepatic granulomas and fibrosis by suppressing NF‐κB signaling and reducing the expression of VEGF, TNF‐α, and MCP‐1. Knowing that NF‐κB pathway can be inhibited by salicylic acid (SA), [9] we are exploring new, more cost‐effective treatments.

In this report, we chose a combination therapy in which microneedling was followed by 30% SSA chemical peel. This combined therapy enhances the penetration depth of SSA. We were unable to obtain histopathological dates as the patients refused invasive examination, but clinical observations showed that lesions completely resolved in two cases and significantly improved in one case, indicating the therapy's high efficacy.

According to our knowledge, this is the first report of chemical peel treating cutaneous nodular lesions following mesotherapy. However, our findings need be validated in more cases through both basic and clinical research.

## Conflicts of Interest

The authors declare no conflicts of interest.

## Data Availability

Research data are not shared.
